# Rare variants in the *ATM *gene and risk of breast cancer

**DOI:** 10.1186/bcr2919

**Published:** 2011-07-25

**Authors:** David E Goldgar, Sue Healey, James G Dowty, Leonard Da Silva, Xiaoqing Chen, Amanda B Spurdle, Mary Beth Terry, Mary J Daly, Saundra M Buys, Melissa C Southey, Irene Andrulis, Esther M John, Kum Kum Khanna, John L Hopper, Peter J Oefner, Sunil Lakhani, Georgia Chenevix-Trench

**Affiliations:** 1 Department of Dermatology, University of Utah School of Medicine, 30 N. 1900 E, Salt Lake City, UT 84132-2101, USA; 2 Queensland Institute of Medical Research, 300 Herston Road, Herston, QLD 4006, Australia; 3 Centre for Molecular, Environmental, Genetic and Analytic Epidemiology, University of Melbourne, Grattan Street, Parkville, VIC 3010, Australia; 4 University of Queensland Centre for Clinical Research and School of Medicine, Building 71/918 RBWH Bowen Bridge Road, Herston, QLD 4029, Australia; 5 Department of Epidemiology, Columbia University, 630 West 168th St, Box 49, New York, NY 10032, USA; 6 Fox Chase Cancer Center, 33 Cottman Ave/100 Laurel Avenue, Philadelphia, PA 19111, USA; 7 Huntsman Cancer Institute, University of Utah, 2000 Circle of Hope, Salt Lake City, UT 84112, USA; 8 Department of Pathology, University of Melbourne, Grattan Street, Parkville, VIC 3010, Australia; 9 Cancer Care Ontario, Fred A. Litwin Center for Cancer Genetics, Samuel Lunenfeld Research Institute, Mount Sinai Hospital, 620 University Avenue, Toronto, Ontario M5G, Canada; 10 Cancer Prevention Institute of California, 2201 Walnut Avenue, Suite 300, Fremont, CA 94538, USA; 11 The Peter MacCallum Cancer Centre, St Andrews Place, East Melbourne, VIC 3002, Australia; 12 Institute of Functional Genomics, University of Regensburg, Josef-Engert-Str.9, 93053, Regensburg, Germany; 13 The Royal Brisbane & Women's Hospital, Cnr Butterfield St and Bowen Bridge Rd, Herston, QLD 4029, Australia

## Abstract

**Introduction:**

The ataxia-telangiectasia mutated (*ATM*) gene (MIM ID 208900) encodes a protein kinase that plays a significant role in the activation of cellular responses to DNA double-strand breaks through subsequent phosphorylation of central players in the DNA damage-response pathway. Recent studies have confirmed that some specific variants in the *ATM *gene are associated with increased breast cancer (BC) risk. However, the magnitude of risk and the subset of variants that are pathogenic for breast cancer remain unresolved.

**Methods:**

To investigate the role of *ATM *in BC susceptibility, we studied 76 rare sequence variants in the *ATM *gene in a case-control family study of 2,570 cases of breast cancer and 1,448 controls. The variants were grouped into three categories based on their likely pathogenicity, as determined by *in silico *analysis and analyzed by conditional logistic regression. Likely pathogenic sequence variants were genotyped in 129 family members of 27 carrier probands (15 of which carried c.7271T > G), and modified segregation analysis was used to estimate the BC penetrance associated with these rare *ATM *variants.

**Results:**

In the case-control analysis, we observed an odds ratio of 2.55 and 95% confidence interval (CI, 0.54 to 12.0) for the most likely deleterious variants. In the family-based analyses, the maximum-likelihood estimate of the increased risk associated with these variants was hazard ratio (HR) = 6.88 (95% CI, 2.33 to 20.3; *P *= 0.00008), corresponding to a 60% cumulative risk of BC by age 80 years. Analysis of loss of heterozygosity (LOH) in 18 breast tumors from women carrying likely pathogenic rare sequence variants revealed no consistent pattern of loss of the *ATM *variant.

**Conclusions:**

The risk estimates from this study suggest that women carrying the pathogenic variant, *ATM *c.7271T > G, or truncating mutations demonstrate a significantly increased risk of breast cancer with a penetrance that appears similar to that conferred by germline mutations in *BRCA2*.

## Introduction

The ataxia-telangiectasia mutated (*ATM*) gene (MIM ID 208900) encodes a protein kinase that plays a major role in activating cellular responses to DNA double-strand breaks through downstream phosphorylation of central players in the DNA damage-response pathways, including BRCA1, p53, and Chk2 [1].

More than 20 years ago, Swift *et al. *[2] reported that female relatives of patients with the autosomal recessive condition, ataxia-telangiectasia (AT), have an elevated risk of cancer, particularly breast cancer. Since the cloning of the *ATM *gene in 1995 [3], many case-control studies have carried out mutation screening and single nucleotide polymorphism (SNP) genotyping to clarify the role of *ATM *genetic variation in breast cancer predisposition [4-10]. Initially, most mutation-screening studies were limited to protein-truncating mutations identified by using the protein-truncating test [11], and many of them were underpowered [12].

The role of *ATM *variants in breast cancer predisposition remained controversial until Renwick *et al. *[13] screened a series of "familial" breast cancer cases selected for having a strong family history and controls unselected for family history of breast cancer. Invoking a multiplicative model in which risk modified a presumed underlying polygenic effect, they estimated that the variants that are known to cause AT in the bi-allelic state confer, on average, a moderately increased risk of breast cancer of about 2.4-fold (95% confidence interval (CI), 1.51 to 3.78) (see also [14]). However, this study did not distinguish between the effects of protein-truncating and missense mutations, although Gatti *et al. *[15] had hypothesised in 1999 that, compared with protein-truncating mutations, some missense variants in *ATM *might act as dominant negatives and confer a particularly high risk of breast cancer when heterozygous, although causing a milder form of AT when homozygous.

To determine which rare missense variants in *ATM *were likely to confer an increased risk of breast cancer, and to compare this with the risk conferred by protein-truncating mutations, we previously carried out a meta-analysis of published data and also mutation screened almost 1,000 breast cancer cases and a similar number of controls [16]. In addition, that study classified the rare missense variants by using an *in silico *missense substitution analysis that provides a ranking of missense variants from evolutionarily most likely (C0) to least likely (C65). We found marginal evidence that protein-truncating (T) and splice-site junction (SJ) mutations confer on average a moderately increased risk of breast cancer (odds ratio (OR), 2.3; 95% CI, 1.1 to 4.8), but stronger evidence that a subset of rare, evolutionarily unlikely missense (rMS) C65 substitutions conferred on average a higher risk of breast cancer (OR, 18; 95% CI, 3 to 120).

To define better the risks associated with these classes of *ATM *variants, and to determine whether they were likely to act as dominant negatives, we genotyped a large panel of rare missense variants, as well as truncating and splice-junction mutations, in breast cancer cases and controls from four large studies. We also genotyped all available relatives of breast cancer cases found to carry putative breast-cancer associated *ATM *variants to estimate their penetrance. In addition, we carried out loss of heterozygosity (LOH) analyses and a review of the pathology of breast tumors from these mutation carriers.

## Materials and methods

### Subjects

We studied Caucasian cases of breast cancer (*n *= 2,517 invasive and 53 DCIS) and controls (*n *= 1,448) from three sources: (a) population-based case and control breast cancer families from the NCI-sponsored Breast Cancer Family Registry (BCFR) [17]; (b) a clinic-based resource of Australian and New Zealand multiple-case breast cancer families from the Kathleen Cuningham Foundation Consortium for Research on Familial Breast Cancer (kConFab) [18]; and (c) Australian female controls chosen from the Red Cross Blood Bank to be ethnically and frequency matched for age to the age at diagnosis of kConFab cases [19] (Table 1). The kConFab cases were those from whom DNA was available who had the youngest age at diagnosis in the family. All subjects in these studies provided informed consent for participation in genetic and family studies. We excluded any subjects who had previously been included in the sequencing study of Tavtigian [16] but noted that some of the included BCFR subjects overlap with those of Bernstein *et al. *[10], although they genotyped only two variants, one of which is in our iPLEX [10]. The individual resource collections (BCFR, kConFab), as well as the specific *ATM *study, have been approved by the relevant ethical committees.

**Table 1:** Numbers of cases and controls by center, after exclusions

Center	Number of cases (DCIS)	Average age at diagnosis	Number of controls	Reference age
Australia BCFR	820 (4)	46	320	45
Ontario BCFR	1,141 (21)	50	591	51
Northern California BCFR	266 (8)	48	377	48
kConFab/RCBB	343 (20)	46	160	46
Total	2,570 (53)	47.9	1,448	48.4

Reference age is defined as age at diagnosis for cases and age at enrollment for controls.

BCFR, Breast Cancer Family Registry; DCIS, ductal carcinoma *in situ*; kConFab, Kathleen Cuningham Foundation Consortium for Research into Familial Breast Cancer.

### Selection of ATM variants and genotyping

Missense variants and in-frame deletions were assessed for the degree of conservation within the ATM multiple protein sequence alignment and for the predicted severity of the amino acid substitution, according to the Align-GVGD class, as previously described [16,20]. We selected all the A-GVGD class C55/C65 variants reported previously [16], as well as a subset of the C0, C15, C25, C35, and C45 variants (Additional file 1). In addition, we included three variants identified in the literature [13,21] and 17 that we had found by sequencing of familial breast cancer cases from the population-based (Northern California, Ontario, Australia) and clinic-based (Philadelphia, New York, Utah) sites of the BCFR (unpublished data). The MassARRAY assay design software (Version 3.1) was used to select oligonucleotide sequences that were best suited for genotyping according to the guidelines of Sequenom Inc San Diego, CA, USA. Sequences are available on request. Primer extension reactions were carried out according to the manufacturer's instructions for iPLEX chemistry. Genotypes were analyzed by using Sequenom TYPER software (Version 3.1). Positive controls for 67 of the 79 variants were included in the iPLEX genotyping. All the rare variants detected by iPLEX and a random selection of the common variants (for QC purposes) were confirmed by direct sequencing by using newly designed PCR primers. In addition, we applied similar QC criteria to those used by the Breast Cancer Association Consortium [22]. Forty-five samples failed QC, but only three of 79 genotyped variants failed QC (ATMc.6067G > A; ATMc.6820G > A; ATMc.8741T > C). We classified the 76 variants into three groups: Group 1 consisted of 36 missense variants with an A-GVGD class of C0 or C15. Group 2 consisted of a total of 18 variants comprising intronic variants (other than those at ± 1 or 2); variants in A-GVGD classes C25, C35, C45; as well as variants in class C55 or C65 that fell outside the FAT and kinase domains of the ATM protein. Group 3 consisted of 22 C55 and C65 variants in the FAT and kinase domains [16], as well as protein-truncating variants (either splice, nonsense, or frameshift).

### Family genotyping and loss of heterozygosity analysis

To estimate the penetrance of the likely deleterious *ATM *variants, 129 family members of women who had been found to carry a truncating mutation (*n *= 10), splice-site variant (*n *= 1), or evolutionarily unlikely missense substitution (C65 and C55) in the FAT, kinase, and FATC domains (*n *= 16) were genotyped for the respective variant by direct sequencing (Table 2). In eight of these families, no additional DNA samples were available, but because they were from population-based sources, they were informative for the penetrance estimation. Twenty-four tumor blocks were available for LOH analysis from 18 different affected cases and female relatives carrying a putative breast-cancer associated variant. Sections were cut, and one slide was stained with hematoxylin and eosin (H&E) and reviewed by a pathologist (LdS). If the section contained at least 70% tumor cells, then the slide from an adjacent unstained section was macro-dissected and DNA isolated [23]. For two cases in which fewer than 70% tumor cells were present in the section, tumor cells were collected by laser capture micro-dissection (LCM) before DNA isolation [24]. Primers that spanned the relevant region were then designed to generate a small PCR product, and the tumor and germline DNA were sequenced in tandem. LOH was scored by the absence of the heterozygous peak seen in the germline sample.

**Table 2:** Characteristics of the families used in the estimation of *ATM *penetrance

Family ID	Variant	Type	Total BC	BC < 50	ATM^+ ^(obligates)	ATM^- ^	No. of Individuals
O01^1 ^	c.170G > A	TSJ	3	2	1	0	21
O02^4 ^	c.1924G > T	TSJ	3	2	2	0	10
K01^1 ^	c.3802delG	TSJ	2	2	2	1	24
N01^4 ^	c.3802delG	TSJ	2	1	1	1	24
A01^5 ^	c.3802delG	TSJ	1	1	0	0	10
O03^5 ^	c.3802delG	TSJ	4	0	0	0	16
K02^1 ^	c.5623C > T	TSJ	6	2	3	11	159
N02^4 ^	c.6997dupA	TSJ	4	0	0	2	15
K03^1,2,3 ^	c.7271T > G	M	5	3	9	7	82
K04^5 ^	c.7271T > G	M	9	6	7	22	162
K05^1 ^	c.7271T > G	M	3	2	1	1	21
K06^1 ^	c.7271T > G	M	8	5	0	13	72
K07^1 ^	c.7271T > G	M	6	4	3	2	38
K08^5 ^	c.7271T > G	M	4	2	3 (3)	4	66
K09^1 ^	c.7271T > G	M	5	4	2	1	36
N03^2 ^	c.7271T > G	M	3	2	0	0	16
O04^5 ^	c.7271T > G	M	3	1	0 (1)	0	18
O05^2 ^	c.7271T > G	M	1	1	0	0	17
O06^5 ^	c.7271T > G	M	2	1	0	0	18
O07^2 ^	c.7271T > G	M	2	0	0	0	19
O08^5 ^	c.7271T > G	M	4	1	0	3	31
O09^2 ^	c.7271T > G	M	5	4	0	0	15
O10^2 ^	c.7271T > G	M	3	1	0	1	23
N04^4 ^	c.7831_7835del	TSJ	5	0	1	1	23
K10^1 ^	c.7886_7890del	TSJ	2	2	0	1	26
O11^5 ^	c.8734A > G	M	5	1	1	1	26
K11^1 ^	c.8851-1G > T	TSJ	8	1	7	14	154
Total			108	51	43 (4)	86	1142

M, missense variant; TSJ, truncating or splice-site mutation.

^1 ^Tavtigian *et al *(2009); ^2 ^Bernstein *et al *(2006); ^3 ^Chenevix-Trench *et al *(2002); ^4 ^found by direct sequencing (unpublished data); ^5 ^found by iPLEX, this study.

### Pathology review

A blinded pathology review was performed by one of us (SRL) on 35 H&E slides of *ATM*-positive breast tumors (from 21 different carriers of Group 3 *ATM *variants) and H&E slides of 38 control breast tumors (age matched within 6 years) ascertained from the Royal Brisbane and Women's Hospital between 2004 and 2009. The slides were scored for pathologic features by using a modified pro forma that was initially developed for studies on the pathology of BRCA-associated cancers. Specifically, we assessed for the presence of *in situ *disease (LCIS and DCIS), invasive tumor type, and overall nuclear grade by using the modified Nottingham Grading System [25], and for the presence of apocrine, "basal" (pushing margins, central acellular or necrotic zones, lymphocytic infiltrates) and squamous differentiation. These features were assessed without ancillary immunohistochemical methods.

### Statistical methods

Conditional logistic regression was used to examine the associations between variants in a given class and the risk of breast cancer, stratified by study center, by using a case-control design. To guard against results driven by individual study centers, we also performed Mantel-Haenszel χ^2 ^analysis comparing each variant group against the reference, stratified by study center. All analyses were performed by using STATA 10.0 (Statcorp, College Station, TX).

Penetrance of *ATM *variants was estimated by using modified segregation analysis of family genotypes adjusted for ascertainment. Models were fitted under maximum likelihood theory by using the statistical package Mendel///version 3.2 [26]. Noncarriers were assumed to be at population risks specific to Australia, Canada, and the United States, with incidence rates taken from cancer registry data obtained from Cancer Incidence in Five Continents, VIII (IARC, Lyon), and hazard ratios (HR, the age-specific breast cancer incidence rate in carriers divided by the relevant population rate) were estimated. Ascertainment was accounted for by conditioning the likelihood of each family on the proband's genotype and phenotype (for population-based families that were selected irrespective of family history) or on all phenotypes and the proband's genotype (for clinic- and population-based families that had been selected because of a family history).

As in Antoniou *et al. *[27,28], a mixed model [29] was used that incorporated the effect of an unmeasured polygenic factor on breast cancer risk in addition to any effect due to the *ATM *variant segregating in the pedigree. *P *Values for the modified segregation analyses were based on the likelihood ratio test and were two-sided. Cumulative risk estimates were calculated from the hazard-ratio estimates as 1 minus the exponential of the cumulative incidence, and the corresponding confidence intervals were calculated by using a parametric bootstrap with 5,000 replications. The model assumed a dominant mode of action of the *ATM *variants on breast cancer risks and a combined allele frequency of 0.001 for the variants in the population.

In separate analyses, we examined the risk associated with these *ATM *variants compared with those associated with *BRCA2*, as estimated by Antoniou *et al*., 2003 [30]. In these analyses, the age-specific HR (by decade) was assumed to be a constant multiple of the Antoniou *et al. *estimate, with cumulative penetrances re-estimated at each trial value of the multiplier. This allowed a similar pattern of age-specific effects as in *BRCA2*, but required estimation of only a single parameter.

## Results

Of the 76 *ATM *variants that passed QC, 29 were observed one or more times in the analyzed set of 2,570 cases and 1,448 controls (Additional file [Supplementary-material S1]). Table 3 shows the distribution of variants and number of cases and controls by group and the results of the logistic regression. Overall, no significant association was found between any variant group and the risk of breast cancer. In particular, we observed an odds ratio of 2.55; 95% CI (0.54 to 12.0) for the Group 3 variants, which included the most likely deleterious missense variants and the truncating variants. No evidence was apparent for any heterogeneity in odds ratios between the four study centers. Inclusion of age into the model did not change the results, nor did exclusion of 163 Ashkenazi Jewish women, who were overrepresented in cases and might have harbored a founder mutation (results not shown). Similarly, exclusion of the 53 DCIS cases had little effect on the results. In addition, to account for individual failed assays (after eliminating those that had failed 16 or more assays), we estimated the probability that a given individual belonged to each group based on the number of failed assays composing that group.

**Table 3:** Breast cancer risk associated with each group of *ATM *variants

Group	Number of Variants	Cases	Controls	Odds Ratio	95% confidence interval
No variant (referent)	-	2,423	1,367	1.0	
Group 1	19	79	45	0.99	0.67-1.45
Group 2	8	59	334	1.10	0.71-1.70
Group 3	3	9	2	2.55	0.54-12.0

Group 1 includes all rare variants classified by the A-GVGD algorithm as C0; Group 3 includes all rare variants that are C55/C65 in either the FAT or kinase domains of the ATM protein as well as all truncating variants and splice variants at the consensus sites. Group 2 includes all other rare variants (see Additional file 1 for details). *ATM*, Ataxia telangiectasia mutated.

### Penetrance analysis in families

**Figure 1:**
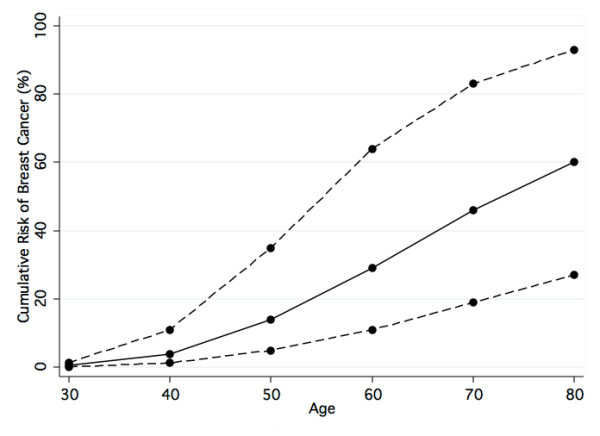
**Penetrance of the *ATM *variants associated with breast cancer risk**. Solid line, Maximum likelihood estimate of cumulative risk of breast cancer; dashed lines, lower and upper 95% confidence limits.

We genotyped additional relatives in all 27 families in which putative pathogenic variants had been identified. The specific variants included 16 missense variants, of which 15 were p.Val2424Gly (c.7271T > G) (rs28904921), seven were frameshifts (of which four were c.3802delG), three were nonsense mutations, and one was a consensus splice-site variant (all variants included in the family analysis are indicated in Table 2). In total, 129 additional DNA samples were available for genotyping in relatives of the probands; 86 were negative for the family-specific variant (10 affected and 76 unaffected individuals), and 43 were positive (14 affected and 29 unaffected individuals). In the analysis of the *ATM *family data by using a mixed model (*ATM *gene plus polygenic background), the presence of an *ATM *variant increased breast cancer risk by an estimated factor of 6.88 (95% CI, 2.33 to 20.3; *P *= 0.00008) and did not depend on age (*P *= 0.9). The estimated cumulative risks of developing breast cancer for female carriers, assuming US SEER incidence rates, are shown in Figure 1. Separate analyses of the 15 families carrying the *ATM *c.7271T > G variant found that this variant increased breast cancer risk by a factor of 8.0 (95% CI, 2.3 to 27.4; *P *= 0.0005) compared with 4.4 (95% CI, 0.70 to 28.1; *P *= 0.053) for families with other variants.

Under the assumption that the penetrance of the *ATM *variants was a constant multiplier of the *BRCA2 *penetrance, the value of the multiplier that resulted in the best fit to the pedigree data was 0.75 (95% CI, 0.33 to 1.50), indicating that the *ATM *alleles segregating in these 27 families were associated with risks equivalent to 75% those of *BRCA2*. In the 16 families with a missense variant, the penetrance estimate was 1.1 that of *BRCA2*, whereas in the 11 families with a truncating or splice junction (TSJ) mutation, the best estimate was 0.3, although this difference was not significant (χ^2 ^= 2.99; *P *= 0.08).

### Loss of heterozygosity analysis

LOH results for the 18 different affected women are summarized in Table 4. Identical LOH results were obtained for all six cases in which two different blocks from the same tumor were tested. Four of the seven cases with a truncating mutation in *ATM *showed loss of the mutant allele, and the remainder showed no LOH. Of the eight cases with the C65 variant, p.Val2424Gly (ATMc.7271T > G), one showed loss of the wild-type, one showed partial loss of the mutant, and the remainder showed no LOH. Of the remaining two cases with C55 or C65 variants, one showed loss of the mutant allele, and the other had no LOH.

**Table 4:** Loss of heterozygosity in breast tumors from carriers of putative breast cancer-associated *ATM *variants

Nucleotide change	Effect	Site	Identifier	LOH
c.170G > A	Protein truncating	Ontario	43115	No LOH
c.442_446 delGACAT	Protein truncating	kConFab	62558	LOH of variant
c.1924G > T	Protein truncating	Ontario	91015	No LOH
c.3802delG	Protein truncating	kConFab	81253	LOH of variant
c.3802delG	Protein truncating	Ontario	43147	No LOH
c.5623C > T	Protein truncating	kConFab	31015	LOH of variant
c.7271T > G	Align GVGD C65	kConFab	40012	No LOH
c.7271T > G	Align GVGD C65	kConFab	40032	LOH of wild type
c.7271T > G	Align GVGD C65	kConFab	40034	No LOH
c.7271T > G	Align GVGD C65	kConFab	70246	No LOH
c.7271T > G	Align GVGD C65	kConFab	60567	No LOH
c.7271T > G	Align GVGD C65	kConFab	20723	No LOH
c.7271T > G	Align GVGD C65	kConFab	51297	50% LOH of variant
c.7271T > G	Align GVGD C65	kConFab	00574	No LOH
c.7638_7646del9	Align GVGD C65	Ontario	91494	No LOH
c.7886_7890delTATTA	Protein truncating	kConFab	31277	LOH of variant
c.8734A > G	Align GVGD C65	Ontario	62131	LOH of variant

*ATM*, ataxia telangiectasia mutated; kConFab, Kathleen Cuningham Foundation Consortium for Research into Familial Breast Cancer; LOH, loss of heterozygosity.

### Pathology review

We compared the ATM-positive tumors with a set of age-matched control breast tumors. No statistically significant difference was noted in overall histologic grade. Looking at the three individual components of grade (tubule formation, nuclear pleomorphism, and mitotic counts), no apparent differences were related to pleomorphism or tubule scores. However, a marginally significant association was seen between the mitotic score (exact *P *= 0.049), largely because of the paucity of *ATM *tumors with a mitotic score of 3 (two of 18 compared with 14 of 34 of the control tumors). This was also supported by analysis of the quantitative count of mitoses per 10 high-power fields, with some suggestion of lower mitotic rates in the *ATM *tumors than in the control tumors (7.9 versus 19.0 mitoses per 10 high-power fields; *P *= 0.02 by *t *test, with Welch correction for unequal variances).

## Discussion

Gatti *et al. *[15] hypothesised in 1999 that, compared with protein-truncating mutations, some missense variants in *ATM *might act as dominant negatives and confer a particularly high risk of breast cancer when heterozygous, while causing a milder form of AT, when homozygous. It was later shown that a missense mutation, *ATM c*.7271T > G (p.Val2424Gly), appears to confer a high risk of breast cancer and to act as a dominant negative [31,32]. This mutation was first identified in a Scottish family with a mild form of AT [33] and subsequently found in an Australian family [31], but initial estimates of the magnitude of risk were imprecise. Screening of nearly 4,000 population-based breast cancer cases for this mutation identified another six carrier families, and, based on their breast cancer family histories, risk for this mutation was estimated to be increased by ninefold (95% CI, 4 to 19) [10].

To refine the risks associated with different classes of *ATM *variants, and to examine the molecular pathologic characteristics of *ATM*-positive tumors, we genotyped 76 rare *ATM *variants in 2,570 breast cancer cases and 1,448 controls. In addition, we genotyped specific variants in the relatives of probands carrying *ATM *variants judged likely to be pathogenic. Because, by definition, we expect variants conferring moderate to high risks of breast cancer to be rare in the general population, traditional case-control studies of even several thousand cases and controls are typically underpowered to detect associations with these sequence variants. We could potentially pool these variants and compare the aggregate frequency of these variants to increase power. However, the power is often reduced because of the inherent heterogeneity of such variants, in which only a minority is associated with increased risk. One strategy to address this problem is to use *in silico *methods to group variants into categories based on their probability of representing variants that are damaging to the normal protein function. Although a number of such methods are available, we used the Align-GVGD that has been applied to a number of genes, including *BRCA1, BRCA2, CHEK2, ATM*, and mismatch-repair genes [20,34]. However, even with this stratification, we still had insufficient power to detect an association with breast cancer with even the most likely pathogenic variants (OR, 2.55 (95% CI, 0.54 to 12.0), although the effect sizes were comparable with those previously reported [16].

As in other studies of *ATM *and breast cancer risk [13,16], the most common pathogenic variant in our study was the *ATM *c.7271T > G (p.Val2424Gly). Renwick *et al. *[13] did not compare the breast cancer risks associated with protein-truncating versus missense mutations in *ATM*. Bernstein *et al. *[10] had previously identified seven carriers of this mutation in the three population-based sites of the BCFR; however, no family members were genotyped in this study. In our study of these same case-control studies, we independently identified the same four mutation carriers from the Ontario BCFR site, as well as the one carrier from the Australian BCFR site. We did not identify the two *ATM c*.7271T > G (p.Val2424Gly) mutation carriers from the Northern California BCFR site that Bernstein et al. [10] had identified because one was subsequently found also to carry a pathogenic *BRCA2 *mutation (and was thus excluded from our study), and for the other, no DNA sample was available for our analyses. This missense *ATM *variant was first reported to be associated with a mild form of AT and might have originated in the Orkney Islands in Scotland and then spread throughout populations with large numbers of Scottish immigrant populations, such as those of Australia. Our analysis of independent samples from four case-control studies provided some support of the observation by Tavtigian *et al. *[31,32] that this mutation (and perhaps other similar missense mutations with dominant-negative activity) confers a higher risk of breast cancer than do protein-truncating mutations.

As a second approach to verifying and characterizing the role of *ATM *sequence variants in breast cancer, we took advantage of the fact that the resources from which the cases were drawn had also included the relatives of those cases, providing us with the ability to genotype both affected and unaffected relatives of cases in which potentially pathogenic variants had been identified. As in Bernstein *et al. *[10], even in cases in which no additional samples were available, the fact that the some of the breast cancer cases analyzed were from the population-based sites of the BCFR allowed us to make inferences based on the observed incidence of cancer in relatives of index cases carrying the specific *ATM *variant. Our analyses of family data in 27 families of carriers of either protein-truncating (*n *= 11) or rare, evolutionarily unlikely, potentially damaging missense mutations (*n *= 16) demonstrated a significantly increased risk of breast cancer with a penetrance that appears similar to that conferred by germline mutations in *BRCA2*. However, even in a study of this size, the confidence intervals are wide. Suggestive evidence also was noted from the family-based analysis that a higher risk was associated with the *ATM *c.7271T > G (p.Val2424Gly) mutation than with truncating mutations, although these differences were not statistically significant because of the relatively small sample size of families. The penetrance associated with truncating mutations was only marginally significant. If our estimates of breast cancer risk are correct, then women carrying the *ATM *c.7271T > G variant would be at sufficiently high risk to warrant screening for at least this variant in multiple-case families without mutations in *BRCA1 *or *BRCA2*. If such a variant is identified, these women could be counseled in a manner similar to that with *BRCA2 *carriers, and those affected with breast cancer might also be candidates for treatment with PARP inhibitors in a manner similar to that with *BRCA1 *and *BRCA2 *carriers. This suggestion is based on the evidence that the inhibition of PARP1 is synthetically lethal with mutation or loss of ATM, and the effect is mediated through mitotic catastrophe independent of apoptosis [35,36].

Consistent with the dominant-negative hypothesis [15,31,32], we did not observe consistent loss of the wild-type allele in tumors from carriers of missense variants. Loss of both the wild-type and the variant allele was observed in different tumors, whereas some tumors from missense carriers exhibited no loss of either allele. Interestingly, we noted that all four carriers of truncating mutations, in which LOH was present, showed loss of the variant, rather than of the wild type, as would be expected for a tumor-suppressor gene. This apparent bias in allelic loss requires further investigation in larger studies.

Blinded pathology review of 35 tumors from cases who carried a likely deleterious *ATM *variant and a hospital-based series of 38 age-matched control breast tumors did not reveal any distinctive pattern of histopathologic characteristics, as had been previously reported in *BRCA1 *tumors [37]. However, some evidence suggested that *ATM *tumors were associated with a lower mitotic index than were control tumors, which is in contrast to the clear increase in mitotic index associated with *BRCA1 *tumors [37]. In agreement with this, our previous expression analysis of six tumors from *ATM *c.7271T > G mutation carriers showed that they were all luminal A or B tumors, and we would not have expected them to share histopathologic characteristics with *BRCA1 *tumors [32]. In contrast to the evidence of Dork *et al. *[12], who reported an increased frequency of lobular breast cancers in *ATM *carriers, we did not observe this in our series, with half the lobular or mixed lobular/ductal in each group (*P *= 0.66).

## Conclusions

This is the largest study to date investigating large numbers of rare missense variants in the *ATM *gene for association with breast cancer risk. In addition to the standard case-control approach, we used the power of a family-based design inherent in the two resources from which the cases and controls were derived (BCFR and kConFab) to estimate more precisely the risks of breast cancer through genotyping of relatives of the probands carrying the putative pathogenic variants. Although Bernstein *et al. *[10] used a similar approach and five probands overlapped between the two studies; it should be noted that, unlike our study, Bernstein *et al. *did not include genotypes of additional relatives from these families in their analysis. Taken as a whole, our study adds to the growing body of evidence that a subset of rare *ATM *variants confers levels of risk that may have clinical implications for the women who carry them, as well as for their at-risk family members.

## Abbreviations

AT: ataxia telangiectasia; *ATM*: ataxia telangiectasia mutated; BC: breast cancer; BCFR: Breast Cancer Family Registry; CI: confidence interval; DCIS: ductal carcinoma *in situ*; H&E: hematoxylin and eosin; HR: hazard ratio; kConFab: Kathleen Cuningham Foundation Consortium for Research into Familial Breast Cancer; LCM: laser capture microdissection; LCIS: lobular carcinoma *in situ*; LOH: loss of heterozygosity; OR: odds ratio; rMS: rare missense; SJ: splice-site junction; T: protein truncating.

## Competing interests

The authors declare that they have no competing interests.

## Authors' contributions

GCT participated in the writing of the manuscript, conceived and designed the study, and directed the laboratory work. DEG and JLH participated in the writing of the manuscript and performed the statistical analyses. SH participated in the writing of the manuscript and performed the iPLEX genotyping and the LOH studies. MCS and EMJ participated in the writing of the manuscript and managed the data. PJO participated in the writing of the manuscript and performed the DNA sequencing. KKK participated in the writing of the manuscript. XC performed the iPLEX genotyping and the LOH studies. JGD performed the statistical analyses. MJD, SB, ABS, IA, and MBT managed the data. SL and LdS performed the pathology review. All authors read and approved the final manuscript.

## Supplementary Material

Additional file 1 ***ATM *variants genotyped in cases and controls**. Click here for file

## References

[B1] Shiloh Y ATM and related protein kinases: safeguarding genome integrity Nat Rev Cancer 2003 3 155 168 10.1038/nrc1011 12612651

[B2] Swift M Reitnauer PJ Morrell D Chase CL Breast and other cancers in families with ataxia-telangiectasia N Engl J Med 1987 316 1289 1294 10.1056/NEJM198705213162101 3574400

[B3] Savitsky K Sfez S Tagle DA Ziv Y Sartiel A Collins FS Shiloh Y Rotman G The complete sequence of the coding region of the ATM gene reveals similarity to cell cycle regulators in different species Hum Mol Genet 1995 4 2025 2032 10.1093/hmg/4.11.2025 8589678

[B4] Fletcher O Johnson N dos Santos Silva I Orr N Ashworth A Nevanlinna H Heikkinen T Aittomaki K Blomqvist C Burwinkel B Missense variants in ATM in 26,101 breast cancer cases and 29,842 controls Cancer Epidemiol Biomarkers Prev 2010 19 2143 2151 10.1158/1055-9965.EPI-10-0374 20826828 PMC2938473

[B5] Izatt L Greenman J Hodgson S Ellis D Watts S Scott G Jacobs C Liebmann R Zvelebil MJ Mathew C Solomon E Identification of germline missense mutations and rare allelic variants in the ATM gene in early-onset breast cancer Genes Chromosomes Cancer 1999 26 286 294 10.1002/(SICI)1098-2264(199912)26:4<286::AID-GCC2>3.0.CO;2-X 10534763

[B6] Teraoka SN Malone KE Doody DR Suter NM Ostrander EA Daling JR Concannon P Increased frequency of ATM mutations in breast carcinoma patients with early onset disease and positive family history Cancer 2001 92 479 487 10.1002/1097-0142(20010801)92:3<479::AID-CNCR1346>3.0.CO;2-G 11505391

[B7] Dork T Bendix R Bremer M Rades D Klopper K Nicke M Skawran B Hector A Yamini P Steinmann D Weise S Stuhrmann M Karstens JH Spectrum of ATM gene mutations in a hospital-based series of unselected breast cancer patients Cancer Res 2001 61 7608 7615 11606401

[B8] Sommer SS Jiang Z Feng J Buzin CH Zheng J Longmate J Jung M Moulds J Dritschilo A ATM missense mutations are frequent in patients with breast cancer Cancer Genet Cytogenet 2003 145 115 120 10.1016/S0165-4608(03)00119-5 12935922

[B9] Thorstenson YR Roxas A Kroiss R Jenkins MA Yu KM Bachrich T Muhr D Wayne TL Chu G Davis RW Wagner TM Oefner PJ Contributions of ATM mutations to familial breast and ovarian cancer Cancer Res 2003 63 3325 3333 12810666

[B10] Bernstein JL Teraoka S Southey MC Jenkins MA Andrulis IL Knight JA John EM Lapinski R Wolitzer AL Whittemore AS West D Seminara D Olson ER Spurdle AB Chenevix-Trench G Giles GG Hopper JL Concannon P Population-based estimates of breast cancer risks associated with ATM gene variants c.7271T > G and c.1066-6T > G (IVS10-6T > G) from the Breast Cancer Family Registry Hum Mutat 2006 27 1122 1128 10.1002/humu.20415 16958054

[B11] FitzGerald MG Bean JM Hegde SR Unsal H MacDonald DJ Harkin DP Finkelstein DM Isselbacher KJ Haber DA Heterozygous ATM mutations do not contribute to early onset of breast cancer Nat Genet 1997 15 307 310 9054948 10.1038/ng0397-307

[B12] Bishop DT Hopper J AT-tributable risks? Nat Genet 1997 15 226 10.1038/ng0397-226 9054927

[B13] Renwick A Thompson D Seal S Kelly P Chagtai T Ahmed M North B Jayatilake H Barfoot R Spanova K McGuffog L Evans DG Eccles D Breast Cancer Susceptibility Collaboration (UK) Easton DF Stratton MR Rahman N ATM mutations that cause ataxia-telangiectasia are breast cancer susceptibility alleles Nat Genet 2006 38 873 875 10.1038/ng1837 16832357

[B14] Stratton MR Rahman N The emerging landscape of breast cancer susceptibility Nat Genet 2008 40 17 22 10.1038/ng.2007.53 18163131

[B15] Gatti RA Tward A Concannon P Cancer risk in ATM heterozygotes: a model of phenotypic and mechanistic differences between missense and truncating mutations Mol Genet Metab 1999 68 419 423 10.1006/mgme.1999.2942 10607471

[B16] Tavtigian SV Oefner PJ Babikyan D Hartmann A Healey S Le Calvez-Kelm F Lesueur F Byrnes GB Chuang SC Forey N Feuchtinger C Gioia L Hall J Hashibe M Herte B McKay-Chopin S Thomas A Vallée MP Voegele C Webb PM Whiteman DC Australian Cancer Study Breast Cancer Family Registries (BCFR) Kathleen Cuningham Foundation Consortium for Research into Familial Aspects of Breast Cancer (kConFab) Sangrajrang S Hopper JL Southey MC Andrulis IL John EM Chenevix-Trench G Rare, evolutionarily unlikely missense substitutions in ATM confer increased risk of breast cancer Am J Hum Genet 2009 85 427 446 10.1016/j.ajhg.2009.08.018 19781682 PMC2756555

[B17] John EM Hopper JL Beck JC Knight JA Neuhausen SL Senie RT Ziogas A Andrulis IL Anton-Culver H Boyd N Buys SS Daly MB O'Malley FP Santella RM Southey MC Venne VL Venter DJ West DW Whittemore AS Seminara D for the Breast Cancer Family Registry The Breast Cancer Family Registry: An infrastructure for cooperative multinational, interdisciplinary and translational studies of the genetic epidemiology of breast cancer Breast Cancer Res 2004 6 R375 R389 10.1186/bcr801 15217505 PMC468645

[B18] Mann GJ Thorne H Balleine RL Butow PN Clarke CL Edkins E Evans GM Fereday S Haan E Gattas M Giles GG Goldblatt J Hopper JL Kirk J Leary JA Lindeman G Niedermayr E Phillips KA Picken S Pupo GM Saunders C Scott CL Spurdle AB Suthers G Tucker K Chenevix-Trench G Kathleen Cuningham Consortium for Research in Familial Breast Cancer Analysis of cancer risk and BRCA1 and BRCA2 mutation prevalence in the kConFab familial breast cancer resource Breast Cancer Res 2006 8 R12 PubMed PMID: 16507150 10.1186/bcr1377 16507150 PMC1413975

[B19] O'Mara TA Fahey P Ferguson K Marquart L Lambrechts D Despierre E Vergote I Amant F Hall P Liu J Czene K SASBAC Rebbeck TR WISE Study Group AOCS Management Group SEARCH Ahmed S Dunning AM Gregory CS Shah M ANECS Webb PM Spurdle AB Progesterone receptor gene variants and risk of endometrial cancer Carcinogenesis 2011 32 331 335 10.1093/carcin/bgq263 21148628 PMC3105584

[B20] Tavtigian SV Greenblatt MS Lesueur F Byrnes GB In silico analysis of missense substitutions using sequence-alignment based methods Hum Mutat 2008 29 1327 1336 10.1002/humu.20892 18951440 PMC3431198

[B21] Concannon P Gatti RA Diversity of ATM gene mutations detected in patients with ataxia-telangiectasia Hum Mutat 1997 10 100 107 10.1002/(SICI)1098-1004(1997)10:2<100::AID-HUMU2>3.0.CO;2-O 9259193

[B22] Cox A Dunning AM Garcia-Closas M Balasubramanian S Reed MW Pooley KA Scollen S Baynes C Ponder BA Chanock S A common coding variant in CASP8 is associated with breast cancer risk Nat Genet 2007 39 352 358 10.1038/ng1981 17293864

[B23] Levi S Urbano-Ispizua A Gill R Thomas DM Gilbertson J Foster C Marshall CJ Multiple K-ras codon 12 mutations in cholangiocarcinomas demonstrated with a sensitive polymerase chain reaction technique Cancer Res 1991 51 3497 3502 1675933

[B24] Espina V Wulfkuhle JD Calvert VS VanMeter A Zhou W Coukos G Geho DH Petricoin EF Liotta LA Laser-capture microdissection Nat Protoc 2006 1 586 603 10.1038/nprot.2006.85 17406286

[B25] Rakha EA Reis-Filho JS Baehner F Dabbs DJ Decker T Eusebi V Fox SB Ichihara S Jacquemier J Lakhani SR Palacios J Richardson AL Schnitt SJ Schmitt FC Tan PH Tse GM Badve S Ellis IO Breast cancer prognostic classification in the molecular era: the role of histological grade Breast Cancer Res 2010 12 207 20804570 10.1186/bcr2607 PMC2949637

[B26] Lange K Weeks D Boehnke M Programs for Pedigree Analysis: MENDEL, FISHER, and dGENE Genet Epidemiol 1988 5 471 472 10.1002/gepi.1370050611 3061869 3061869

[B27] Antoniou AC Pharoah PD McMullan G Day NE Ponder BA Easton D Evidence for further breast cancer susceptibility genes in addition to BRCA1 and BRCA2 in a population-based study Genet Epidemiol 2001 21 1 18 10.1002/gepi.1014 11443730

[B28] Antoniou AC Spurdle AB Sinilnikova OM Healey S Pooley KA Schmutzler RK Versmold B Engel C Meindl A Arnold N Common breast cancer-predisposition alleles are associated with breast cancer risk in BRCA1 and BRCA2 mutation carriers Am J Hum Genet 2008 82 937 948 10.1016/j.ajhg.2008.02.008 18355772 PMC2427217

[B29] Thompson EA Cannings C Skolnick MH Ancestral inference. I. The problem and the method Ann Hum Genet 1978 42 95 108 10.1111/j.1469-1809.1978.tb00934.x 686688

[B30] Antoniou A Pharoah PD Narod S Risch HA Eyfjord JE Hopper JL Loman N Olsson H Johannsson O Borg A Average risks of breast and ovarian cancer associated with BRCA1 or BRCA2 mutations detected in case series unselected for family history: a combined analysis of 22 studies Am J Hum Genet 2003 72 1117 1130 10.1086/375033 12677558 PMC1180265

[B31] Chenevix-Trench G Spurdle AB Gatei M Kelly H Marsh A Chen X Donn K Cummings M Nyholt D Jenkins MA Scott C Pupo GM Dörk T Bendix R Kirk J Tucker K McCredie MR Hopper JL Sambrook J Mann GJ Khanna KK Dominant negative ATM mutations in breast cancer families J Natl Cancer Inst 2002 94 205 215 11830610 10.1093/jnci/94.3.205

[B32] Waddell N Jonnalagadda J Marsh A Grist S Jenkins M Hobson K Taylor M Lindeman GJ Tavtigian SV Suthers G Goldgar D Oefner PJ kConFab Investigators Taylor D Grimmond S Khanna KK Chenevix-Trench G Characterization of the breast cancer associated ATM 7271T > G (V2424G) mutation by gene expression profiling Genes Chromosomes Cancer 2006 45 1169 1181 10.1002/gcc.20381 17001622

[B33] Stankovic T Kidd AM Sutcliffe A McGuire GM Robinson P Weber P Bedenham T Bradwell AR Easton DF Lennox GG Haites N Byrd PJ Taylor AM ATM mutations and phenotypes in ataxia-telangiectasia families in the British Isles: expression of mutant ATM and the risk of leukemia, lymphoma, and breast cancer Am J Hum Genet 1998 62 334 345 10.1086/301706 9463314 PMC1376883

[B34] Arnold S Buchanan DD Barker M Jaskowski L Walsh MD Birney G Woods MO Hopper JL Jenkins MA Brown MA Tavtigian SV Goldgar DE Young JP Spurdle AB Classifying MLH1 and MSH2 variants using bioinformatic prediction, splicing assays, segregation, and tumor characteristics Hum Mutat 2009 30 757 770 10.1002/humu.20936 19267393 PMC2707453

[B35] Bryant HE Helleday T Inhibition of poly (ADP-ribose) polymerase activates ATM which is required for subsequent homologous recombination repair Nucleic Acids Res 2006 34 1685 1691 10.1093/nar/gkl108 16556909 PMC1410911

[B36] Weston VJ Oldreive CE Skowronska A Oscier DG Pratt G Dyer MJ Smith G Powell JE Rudzki Z Kearns P Moss PA Taylor AM Stankovic T The PARP inhibitor olaparib induces significant killing of ATM deficient lymphoid tumour cells in vitro and in vivo Blood 2010 116 4578 4587 10.1182/blood-2010-01-265769 20739657

[B37] Lakhani SR Reis-Filho JS Fulford L Penault-Llorca F van der Vijver M Parry S Bishop T Benitez J Rivas C Bignon YJ Prediction of BRCA1 status in patients with breast cancer using estrogen receptor and basal phenotype Clin Cancer Res 2005 11 5175 5180 10.1158/1078-0432.CCR-04-2424 16033833

