# Supplementary material for: Rare variants in the ATM gene and risk of breast cancer
Source: Breast Cancer Res. 2011 Jul 25;13(4):R73. doi: 10.1186/bcr2919 (PMC3236337; doi:10.1186/bcr2919)
Supplement: Additional file 1 — ATM variants genotyped in cases and controls. [file bcr2919-S1.DOC]

Supplementary Table S1: *ATM* variants genotyped in cases and controls

| **EXON** | **Nucleotide change** | **Amino acid change** | **Effect** | **ATM domain** | **Group** | **Reason for inclusion in iPLEX** |
| --- | --- | --- | --- | --- | --- | --- |
| 5 | c.146C>G | p.Ser49Cys | Align GVGD C15 | p53 & BRCA1 interaction | 1 | BCFR mutation screening |
| 5 | c.170G>A^&^ | p.Trp57X | Protein- truncating | p53 & BRCA1 interaction | 3 | Tavtigian et al. AJHG [2009) 85:1–20 |
| 7 | c.442_446 delGACAT | p.Asp148SerfsX9 | Protein-truncating | p53 & BRCA1 interaction | 3 | Tavtigian et al. AJHG [2009) 85:1–20 |
| 11 | c.1229T>C | p.Val410Ala | Align GVGD C0 |  | 1 | Tavtigian et al. AJHG [2009) 85:1–20 |
| 12 | c.1541G>A | p.Gly514Asp | Align GVGD C0 |  | 1 | Tavtigian et al. AJHG [2009) 85:1–20 |
| 14 | c.1810C>T | p.Pro604Ser | Align GVGD C0 |  | 1 | Tavtigian et al. AJHG [2009) 85:1–20 |
| 14/15 | c.1899-55T>G |  | Intronic |  | 2 | Concannon et al, Cancer Res. [2008) 68(16):6486-91 |
| 15 | c.1924G>T^&^ | p.Glu642X | Protein- truncating |  | 3 | BCFR mutation screening |
| 17 | c.2275A>G | p.Ser759Gly | Align GVGD C0 |  | 1 | BCFR mutation screening |
| 17 | c.2362A>C | p.Ser788Arg | Align GVGD C0 |  | 1 | Tavtigian et al. AJHG [2009) 85:1–20 |
| 18 | c.2442C>A | p.Asp814Glu | Align GVGD C0 | beta-adaptin interaction | 1 | Tavtigian et al. AJHG [2009) 85:1–20 |
| 19 | c.2572T>C | p.Phe858Leu | Align GVGD C0 | beta-adaptin interaction | 1 | BCFR mutation screening |
| 19 | c.2614C>T | p.Pro872Ser | Align GVGD C0 | beta-adaptin interaction | 1 | Tavtigian et al. AJHG [2009) 85:1–20 |
| 22 | c.2932T>C | p.Ser978Pro | Align GVGD C65 | beta-adaptin interaction | 2 | Tavtigian et al. AJHG [2009) 85:1–20 |
| 23 | c.3131A>G | p.Asn1044Ser | Align GVGD C0 | beta-adaptin interaction | 1 | BCFR mutation screening |
| 24 | c.3161C>G | p.Pro1054Arg | Align GVGD C65 | beta-adaptin interaction | 2 | BCFR mutation screening |
| 25 | c.3295G>A | p.Asp1099Asn | Align GVGD C0 | beta-adaptin interaction | 1 | Tavtigian et al. AJHG [2009) 85:1–20 |
| 25 | c.3349C>T | p.Gln1117X | Protein truncating | beta-adaptin interaction | 3 | Tavtigian et al. AJHG [2009) 85:1–20 |
| 25 | c.3383A>G | p.Gln1128Arg | Align GVGD C0 | beta-adaptin interaction | 1 | Tavtigian et al. AJHG [2009) 85:1–20 |
| 26 | c.3467C>T | p.Thr1156Met | Align GVGD C0 | beta-adaptin interaction | 1 | Tavtigian et al. AJHG [2009) 85:1–20 |
| 27 | c.3665T>C | p.Leu1222Pro | Align GVGD C65 | beta-adaptin interaction | 2 | BCFR mutation screening |
| 28 | c.3802delG^&^ | p.Val1268X | Protein- truncating | beta-adaptin interaction | 3 | Tavtigian et al. AJHG [2009) 85:1–20 |
| 28 | c.3925G>A | p.Ala1309Thr | Align GVGD C0 |  | 1 | Tavtigian et al. AJHG [2009) 85:1–20 |
| 28/29 | c.3993+5G>T |  | Intronic |  | 2 | BCFR mutation screening |
| 29 | c.4060C>A | p.Pro1354Thr | Align GVGD C0 |  | 1 | Tavtigian et al. AJHG [2009) 85:1–20 |
| 29 | c.4066A>G | p.Asn1356Asp | Align GVGD C0 |  | 1 | Tavtigian et al. AJHG [2009) 85:1–20 |
| 30 | c.4138C>T | p.His1380Tyr | Align GVGD C0 | c_Abl interaction | 1 | Tavtigian et al. AJHG [2009) 85:1–20 |
| 30 | c.4148C>T | p.Ser1383Leu | Align GVGD C65 |  | 2 | Tavtigian et al. AJHG [2009) 85:1–20 |
| 31 | c.4258C>T | p.Leu1420Phe | Align GVGD C0 |  | 1 | BCFR mutation screening |
| 31 | c.4279G>A | p.Ala1427Thr | Align GVGD C0 |  | 1 | Tavtigian et al. AJHG [2009) 85:1–20 |
| 31 | c.4324T>C | p.Tyr1442His | Align GVGD C65 |  | 2 | Tavtigian et al. AJHG [2009) 85:1–20 |
| 31 | c.4388T>G | p.Phe1463Cys | Align GVGD C55 |  | 2 | Tavtigian et al. AJHG [2009) 85:1–20 |
| 31 | c.4414T>G | p.Leu1472Val | Align GVGD C15 |  | 1 | BCFR mutation screening |
| 35 | c.4949A>G | p.Asn1650Ser | Align GVGD C0 |  | 1 | Tavtigian et al. AJHG [2009) 85:1–20 |
| 37 | c.5186T>C | p.Val1729Ala | Align GVGD C25 |  | 2 | Tavtigian et al. AJHG [2009) 85:1–20 |
| 37 | c.5228C>T | p.Thr1743Ile | Align GVGD C65 |  | 2 | Tavtigian et al. AJHG [2009) 85:1–20 |
| 39 | c.5557G>A | p.Asp1853Asn | Align GVGD C15 |  | 1 | BCFR mutation screening |
| 39 | c.5558A>T | p.Asp1853Val | Align GVGD C15 |  | 1 | Tavtigian et al. AJHG [2009) 85:1–20 |
| 39 | c.5623C>T^&^ | p.Arg1875X | Protein- truncating |  | 3 | Tavtigian et al. AJHG [2009) 85:1–20 |
| 41 | c.5821G>C | p.Val1941Leu | Align GVGD C0 |  | 1 | Tavtigian et al. AJHG [2009) 85:1–20 |
| 41 | c.5890A>G | p.Lys1964Glu | Align GVGD C0 |  | 1 | Tavtigian et al. AJHG [2009) 85:1–20 |
| 42 | c.5938G>A | p.Gly1980Arg | Align GVGD C0 |  | 1 | BCFR mutation screening |
| 43 | c.6088A>G | p.Ileu2030Val | Align GVGD C0 |  | 1 | Tavtigian et al. AJHG [2009) 85:1–20 |
| 44 | c.6115G<A | p.Glu2039Lys | Align GVGD C55 |  | 2 | Tavtigian et al. AJHG [2009) 85:1–20 |
| 45 | c.6235G>A | p.Val2079Ile | Align GVGD C0 |  | 1 | Tavtigian et al. AJHG [2009) 85:1–20 |
| 45 | c.6314G>C | p.Arg2105Thr | Align GVGD C65 | FAT | 3 | Tavtigian et al. AJHG [2009) 85:1–20 |
| 45 | c.6343G>A | p.Val2115Ile | Align GVGD C0 | FAT | 1 | BCFR mutation screening |
| 45/46 | c.6348-54T>C |  | Intronic |  | 2 | Concannon et al. Cancer Res. [2008) 68:6486-91 |
| 49 | c.6860G>C | p.Gly2287Ala | Align GVGD C55 | FAT | 3 | Tavtigian et al. AJHG [2009) 85:1–20 |
| 49 | c.6919C>T | p.Leu2307Phe | Align GVGD C0 | FAT | 1 | Tavtigian et al. AJHG [2009) 85:1–20 |
| 50 | c.6995T>C | p.Leu2332Pro | Align GVGD C0 | FAT | 1 | Tavtigian et al. AJHG [2009) 85:1–20 |
| 50 | c.6997dupA^&^ | p.Thr2333AsnfsX40 | Protein truncating | FAT | 3 | Tavtigian et al. AJHG [2009) 85:1–20 |
| 51 | c.7187C>G | p.Thr2396Ser | Align GVGD C0 | FAT | 1 | Tavtigian et al. AJHG [2009) 85:1–20 |
| 51 | c.7202T>C | p.Ile2401Thr | Align GVGD C65 | FAT | 3 | Tavtigian et al. AJHG [2009) 85:1–20 |
| 51 | c.7271T>G^&^ | p.Val2424Gly | Align GVGD C65 | FAT | 3 | Tavtigian et al. AJHG [2009) 85:1–20 |
| 52 | c.7355T>C | p.Leu2452Pro | Align GVGD C25 | FAT | 2 | BCFR mutation screening |
| 52 | c.7357C>T | p.Arg2453Cys | Align GVGD C15 | FAT | 1 | BCFR mutation screening |
| 53 | c.7592T>C | p.Met2531Thr | Align GVGD C0 |  | 1 | Tavtigian et al. AJHG [2009) 85:1–20 |
| 54 | c.7638_7646del9 | p.SRI2546_2548del3 | Align GVGD C65 |  | 2 | Tavtigian et al. AJHG [2009) 85:1–20 |
| 54 | c.7775C>G | p.Ser2592Cys | Align GVGD C65 |  | 2 | Tavtigian et al. AJHG [2009) 85:1–20 |
| 55 | c.7831_7835delAGTAG | p.Ser2611GlufsX9 | Protein- truncating |  | 3 | BCFR mutation screening |
| 55 | c.7886_7890delTATTA^&^ | p.Ile2629SerfsX25 | Protein- truncating |  | 3 | Tavtigian et al. AJHG [2009) 85:1–20 |
| 56 | c.7942C>T | p.Pro2648Ser | Align GVGD C25 |  | 2 | Tavtigian et al. AJHG [2009) 85:1–20 |
| 57 | c.8080G>T | p.Gly2694X | Protein- truncating |  | 3 | BCFR mutation screening |
| 57 | c.8125G>A | p.Gly2709Ser | Align GVGD C55 |  | 2 | Tavtigian et al. AJHG [2009) 85:1–20 |
| 59 | c.8293G>A | p.Gly2765Ser | Align GVGD C55 | kinase | 3 | Tavtigian et al. AJHG [2009) 85:1–20 |
| 59 | c.8314G>A | p.Gly2772Arg | Align GVGD C65 | kinase | 3 | Tavtigian et al. AJHG [2009) 85:1–20 |
| 60 | c.8565_8566TG>AA | p.SV2855_2856RI | Align GVGD C65 | kinase | 3 | Renwick et al., Nature Genetics [2006) 38:873-875 |
| 61 | c.8659C>G | p.His2887Asp | Align GVGD C65 | kinase | 3 | Tavtigian et al. AJHG [2009) 85:1–20 |
| 62 | c.8672G>A | p.Gly2891Asp | Align GVGD C65 | kinase | 3 | Tavtigian et al. AJHG [2009) 85:1–20 |
| 62 | c.8734A>G^&^ | p.Arg2912Gly | Align GVGD C65 | kinase | 3 | Tavtigian et al. AJHG [2009) 85:1–20 |
| 62 | c.8773G>A | p.Gly2925Ser | Align GVGD C55 | kinase | 3 | Tavtigian et al. AJHG [2009) 85:1–20 |
| 64 | c.8851-1G>T^&^ |  | Splice-site | kinase | 3 | Tavtigian et al. AJHG [2009) 85:1–20 |
| 64 | c.8895G>C | p.Leu2965Phe | Align GVGD C0 |  | 1 | BCFR mutation screening |
| 65 | c.9008A>G | p.Asn3003Ser | Align GVGD C45 |  | 2 | Tavtigian et al. AJHG [2009) 85:1–20 |
| 65 | c.9086G>A | p.Gly3029Asp | Align GVGD C15 | FATC | 1 | Tavtigian et al. AJHG [2009) 85:1–20 |

^&^ ATM variant included in family-based analyses.
